# Sum of parts is greater than the whole: inference of common genetic history of populations

**DOI:** 10.1186/1471-2164-14-S1-S10

**Published:** 2013-01-21

**Authors:** Filippo Utro, Marc Pybus, Laxmi Parida

**Affiliations:** 1 Computational Genomics, IBM T J Watson Research, Yorktown, USA; 2 Institute of Evolutionary Biology (CSIC-UPF), Dr. Aiguader 88, 08003 Barcelona, Spain

## Abstract

**Background:**

Reconstructability of population history, from genetic information of extant individuals, is studied under a simulation setting. We do not address the issue of accuracy of the reconstruction algorithms: we assume the availability of the theoretical best algorithm. On the other hand, we focus on the fraction (1 - *f*) of the common genetic history that is irreconstructible or impenetrable. Thus the fraction, *f*, gives an upper bound on the extent of estimability. In other words, there exists no method that can reconstruct a fraction larger than *f *of the entire common genetic history. For the realization of such a study, we first define a natural measure of the amount of genetic history. Next, we use a population simulator (from literature) that has at least two features. Firstly, it has the capability of providing samples from different demographies, to effectively reflect reality. Secondly, it also provides the underlying relevant genetic history, captured in its entirety, where such a measure is applicable. Finally, to compute *f*, we use an information content measure of the relevant genetic history. The simulator of choice provided the following demographies: Africans, Europeans, Asians and Afro-Americans.

**Results:**

We observe that higher the rate of recombination, lower the value of *f*, while *f *is invariant over varying mutation rates, in each of the demographies. The value of *f *increases with the number of samples, reaching a plateau and suggesting that in all the demographies at least about one-third of the relevant genetic history is impenetrable. The most surprising observation is that the the sum of the reconstructible history of the subsegments is indeed larger than the reconstructible history of the whole segment. In particular, longer the chromosomal segment, smaller the value of *f*, in all the demographies.

**Conclusions:**

We present the very first framework for measuring the fraction of the relevant genetic history of a population that is mathematically elusive. Our observed results on the tested demographies suggest that it may be better to aggregate the analysis of smaller chunks of chromosomal segments than fewer large chunks. Also, no matter the richness of samples in a population, at least one-third of the population genetic history is impenetrable. The framework also opens up possible new lines of investigation along the following. Given the characteristics of a population, possibly derived from observed extant individuals, to estimate the (1) optimal sample size and (2) optimal sequence length for the most informative analysis.

## Background

Every genetic event that is consequential to the genetic landscape of a population is captured in a topological structure called the Ancestral Recombinations Graph (ARG) [1]. The converse of this may not hold, that is every genetic event captured in the ARG may not contribute to the observed genetic patterns in the extant population, but nevertheless is a legitimate component of the relevant and common genetic history of the population. In a sense, the ARG is the phylogeny of the individuals of the population. It should be noted that just as in a phylogeny topology, an ARG also does not have any extraneous nodes. Further, it is not unreasonable to assume that there exists a "true" ARG for a collection of samples or a population. Recall that the nodes of the ARG represent genetic events. The topology is not necessarily a tree, due to genetic exchange events such as recombinations, gene duplications and so on. These are represented as nodes with multiple incoming edges in the ARG. The edges are usually annotated with mutation and the lengths are representative of the ages in generation. Thus the topology, together with its annotation and the edge lengths, determines the genetic landscape of the extant samples. The reader is directed to [2] for an exposition on random graph representation of the ARG.

In this paper, we simply use the expected number of nodes in the ARG as a measure of the relevant genetic history of the population. While this may not be precise, it is a fair proxy for the amount of the relevant genetic history. Then a well-defined question to ask is: What is the largest fraction, *f*, of the history that is estimable from a given sample? In other words, no matter what methodological ingenuity is employed, there is always (1 - *f*) fraction of the common history that is impenetrable. Let *N *be the number of nodes in an underlying ARG topology. Given the extant samples, let a method estimate *N*' ≤ *N *nodes. We further assume that the lengths of the edges, as well as the interconnectivity with the labels, are estimated correctly, so the estimated fraction of this ARG, defined as 0.0 ≤ *N*'/*N *≤ 1.0, is a natural "overstimate". Let ${N^{\prime }}_{max}$ be the maximum of ${N^{\prime }}_{i}$ from *all *possible methods *i*. Then *f*, the penetrable fraction, is ${N^{\prime }}_{max}/N$. However, it is impossible to enumerate all possible estimation methods. So, we resort to the mathematical structure called the minimal descriptor [3] of an ARG: it is an essential substructure of an ARG that preserves the genetic landscape of the extant samples, including the topology and edge lengths of the marginal trees. The reader is directed to [4] for an exposition on this nonredundant information content of an ARG. The minimal descriptor is also an ARG and let the number of nodes in the minimal descriptor be Ñ , then ${N^{\prime }}_{max}\leq Ñ$. Thus, this methodology-independent scheme gives an upper bound on the penetrable fraction *f *as Ñ/N, and a lower bound on the impenetrable fraction as 1-Ñ/N.

In this paper, we seek the value of *f*, in human populations. Such a study of attempting to "know the unknowable" is best done in a simulation setting. From literature, we pick a population simulator that has the capability of providing not only individuals from different demographies, but also the underlying ARG. This is very suitable for our experimental set-up. Next, we design an algorithm to extract the minimal descriptor from a given ARG. Thus we compute the upper bound on *f*, as discussed. Recall that each node of the ARG has a specific *age *or *depth *associated with it. It may be noted that the length attribute of an edge can be viewed simply as the non-negative difference between the depths of the two incident nodes. The terminal leafnode are the extant individuals. The depth of the extant individual is defined to be zero and the value progressively increases as one traverses the ARG away from the terminal leaf nodes. The nodes of the minimal descriptor are also the nodes of the underlying ARG and the same age is associated with them. Let *epoch d *be defined as a range of depths say [*d*_1_, *d*_2_] with *d*_2 _≥ *d*_1_. Then the *history density *at *d, N_d_*, is measured by the number of nodes in the ARG with depth in the epoch *d*. Extending this notion, the *estimable density *at *d *is measured as $f_{d}={Ñ}_{d}/N_{d}$, where ${Ñ}_{d}$ is the number of nodes in the minimal descriptor with depth in the epoch *d*. We study the demography characteristics in terms of the history density and the estimable density.

Let tARG denote the true ARG for a given data set with *N *nodes. Then, Ñ  is the number of estimable nodes of tARG, irrespective of the reconstructability of the interconnecting edges. Thus Ñ  is an overestimate and (N-Ñ) is an underestimate of the true values.

### Simulating the populations

We use COSI [5] that is the only population simulator, to the best of our knowledge, that provides the ARG as well as produces populations that match the the genetic landscape of the observed human populations. We use the *bestfit model *in COSI to simulate the samples with a calibrated human demography for different populations, proposed by Schaffner et al. [5]. This demography generates data matching three structured continental populations: Africans, Europeans and Asians. An admixed population, the Afro-Americans, can also be generated. This simulator has also been used in literature as a gold standard for generating the demographies [6-9]. In order to explore *f *and the impenetrable fraction (1 - *f*) of a given demography, all combinations of four different simulation parameters have been used: mutation rate, sequence length, sample size and recombination rate. These are briefly described below:

- mutation rate: According to different studies *Homo sapiens*, as a species, has a mutation rate around 1.5 × 10^-8 ^per base pair per generation (bp/gen for short) [10]. However, this value could change along the genome.

- sequence length: When simulating genetic population data, sequence length is one of the most important factors. While it may not computationally feasible to simulate a whole chromosome, enough polymorphisms are required in order to get meaningful results.

- sample size: The sample size needs to be large enough to capture important population features.

- recombination rate: The mean recombination rate along the genome in *Homo sapiens *is around 1.3 cM/Mb [11]. However it has been seen that it can vary widely in a fine-scale manner when focusing on specific regions of the genome [12]. Different simulations are run using recombination rates matching the major portion of the range observed in human data [5].

Based on the above we used different parameters values, and all possible combinations of them, in order to assess the their effects on *f *(see Table 1).

**Table 1:** Experimental set-up

Parameters	Values
Mutation rate (bp/gen × 10^-8^)	0.7, 1.5, 3.0
Sequence length (Kb)	5, 10, 30, 50, 75, 100, 150, 200
Sample size	5, 10, 30, 60, 120
Recombination rate (cM/Mb)	0.1, 0.3, 0.5, 0.7, 0.9, 1.1, 1.3, 1.5, 1.7, 1.9, 2.1, 2.3, 2.5, 2.8, 3.1, 3.5, 3.9, 4.5, 5.1

Each population of the COSI demography has been tested independently as well as the whole human demography (i.e. all populations together). In total, more than 22800 simulations replicates were generated combining different values for the four simulation parameters described above. This includes ten replicates for each experiment. For the highest value of sequence length used (i.e. 200 Kb), some experiments were terminated after thirty minutes, since no substantial progress was being made towards its completion. Therefore, the results for 200 Kb sequence length are not reported in the summary plots.

## Method

Recall that an ARG is a phylogenetic structure that encodes both duplication events, such as mutations, as well as genetic exchange events, such as recombinations: this captures the (genetic) dynamics of a population evolving over generations. From a topological point of view, an ARG is always a directed acyclic graph where the direction of the edges is toward the more recent generation. An edge is annotated with the mutation genetic event, possible multiple events. Some simulators may give edges with empty labels. Recall that the *length *of the edge, not to be confused with the edge label, represents the epoch defined by the age (or depth) of the two incident nodes. A chain has a single incoming edge and a single outgoing edge. In the ARG we define the following nodes: (a) the *leaf *nodes have no outgoing edges and they represent the extant unit (b) the *coalescent *nodes have single incoming edges, and (c) the *exchange *nodes have multiple incoming edges. Finally, given two nodes *v *and *w*, if there is an outgoing edge from *v *to *w*, then *v *is referred to as *parent *of *w *and *w *is referred as a *child *of *v*.

In [3] a structure-preserving and samples-preserving core of an ARG *G*, called the minimal descriptor ARG (mdARG) of *G *was identified. Its structure-preserving characteristic ensures that the topology and the all the branch lengths of the marginal trees of the minimal descriptor ARG are identical to that of *G *and the samples-preserving property asserts that the patterns of genetic variation in the samples of the minimal descriptor ARG are exactly the same as that of *G*. It was also shown that an unbounded *G *has a finite minimal descriptor, that continues to preserve critical graph-theoretic properties of *G*. Thus this lossless and bounded structure is well defined for all ARGs (including unbounded ARGs) and we use the same here. However, a minimal descriptor of an ARG may not be unique. This does not affect the estimation of *f*, since $N_{max}\leq Ñ\;$, for all possible Ñ  corresponding the the different minimal descriptors (see the Background section for the definitions).

### Identifying the estimable fraction (mdARG)

**Figure 1:**
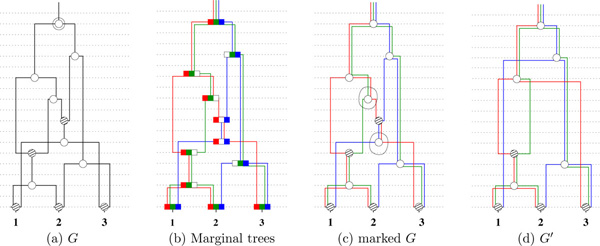
**The topology of an ARG**. (a) The topology of an ARG *G *with three extant samples marked 1, 2 and 3. The dashed horizontal lines mark the age or depth of the nodes which are the same in all the four figures. (b) The three nonmixing segments are red, green and blue, in that order. Each node displays the nonmixing segments. A white rectangle indicates the absence of that segment in that node. The three embedded trees, corresponding to each segment, are shown in the same color as that of the segment. The edge labels (mutation events) are not shown to avoid clutter. (c) The same as (b) with the two marked nodes that are not t-coalescent. (d) Removing the marked nodes to obtain a minimal descriptor *G*'.

This is done by computing a minimal descriptor from the ARG. The input to this process is the ARG *G *derived from the log files of the population simulator COSI. The sequence length is normalized to the interval 0 [1]. This ARG is preprocessed as follows. Firstly, the number of marginal trees, *M*, is extracted from *G*, corresponding to the *M *intervals [0, *l*_1_], [*l*_1_, *l*_2_], .., [*l_M_*- 1, *l_M _*= 1.0] derived from the segments file of COSI, where 0 <*l*_1 _<*l*_2 _< .. <*l_M _*= 1.0. Next, each node in *G *is annotated with one or more of these *M *intervals by traversing *G *appropriately. See Figures 1(a) and 1(b).

**Figure 2:**
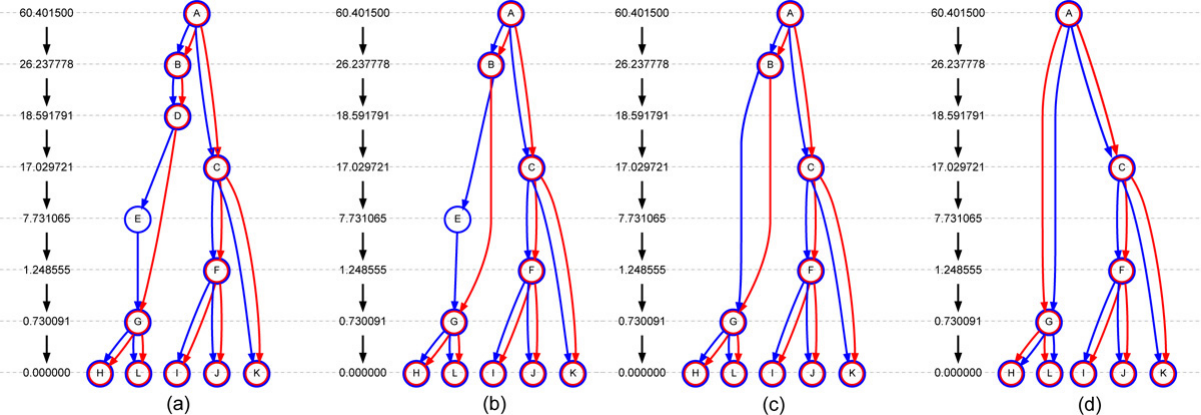
**A simple example using the output of COSI**. A simple example using the output of COSI, where the horizontal line corresponds to the age or depth of the node that it intersects. Also, the *length *of each edge is not proportional to the size in the rendering. For instance the length of edge AB is 34.163732 while length of BD is only 7.645987, while they have been rendered here with the same size. (a) The topology of the ARG: node D is not t-coalescent. (b) The ARG in (a) after removal of node D. (c) The ARG in (b) after removal of chain node E. (d) The ARG in (c) after removal of chain node B.

Recall from [3] that a coalescent node is *t-coalescent *if it is a coalescent node in one of the *M *marginal trees. Each coalescent node that is not t-coalescent is removed, following the node-removal procedure defined in [3]. To remove node *v*, an edge is introduced from the parent, *u*, of *v *to each child *w *of *v*, while maintaining the same age of *u *as well as the child *w*. Also the annotation of the edges is adjusted to reflect the same flow of genetic material from *u *to each of *w*. Based on this, an mdARG is constructed in the following steps. (1) Remove the coalescent nodes that are not t-coalescent, using the node-removal procedure. See Figure 1(d) for an example. Since a coalescent node could also be a recombination node, it is possible that such a node is additionally not t-coalescent. In that case, the node continues to belong to the minimal descriptor. (2) The last step is applied till it is no longer applicable. (3) The chain nodes are removed. Figure 2 provides an example, on a *G *given by COSI, of the above procedure. In particular, given the ARG in Figure 2(a), the node D is not t-coalescent node and then it is removed producing the Figure 2(b). Finally, in Figures 2(c)-(d) step 3 is performed removing the nodes E and B. Let the resulting graph be *G*', which is an mdARG. Let *N *be the number of nodes in *G *and Ñ  the number of nodes in *G*'. Then, f≤Ñ/N.

## Results and discussion

Given the genetic landscape of some extant samples, its underlying ARG is a plausible explanation of the observation, since it is the annotated topological structure that captures the genetic history in its totality, that is relevant to the extant samples. It can also be viewed as a generator that faithfully produces the genetic landscape of the different demographies and since it is a random graph [13], we use multiple replicates to study its the characteristics. We observe that the mutation rate does not influence *f *significantly. Hence, we use a fixed mutation rate of 1.5 × 10^-8 ^bp/gen in the figures. The interested reader is directed to the Additional File 1 for the plots of each experiment for all the other mutation rates. Even though the ARG is not a tree, the density (i.e, number of nodes per epoch) of the relevant genetic events decrease exponentially with depth. Also, the shape of the profiles for the different demographies is independent of the four classes of parameters.

**Figure 3:**
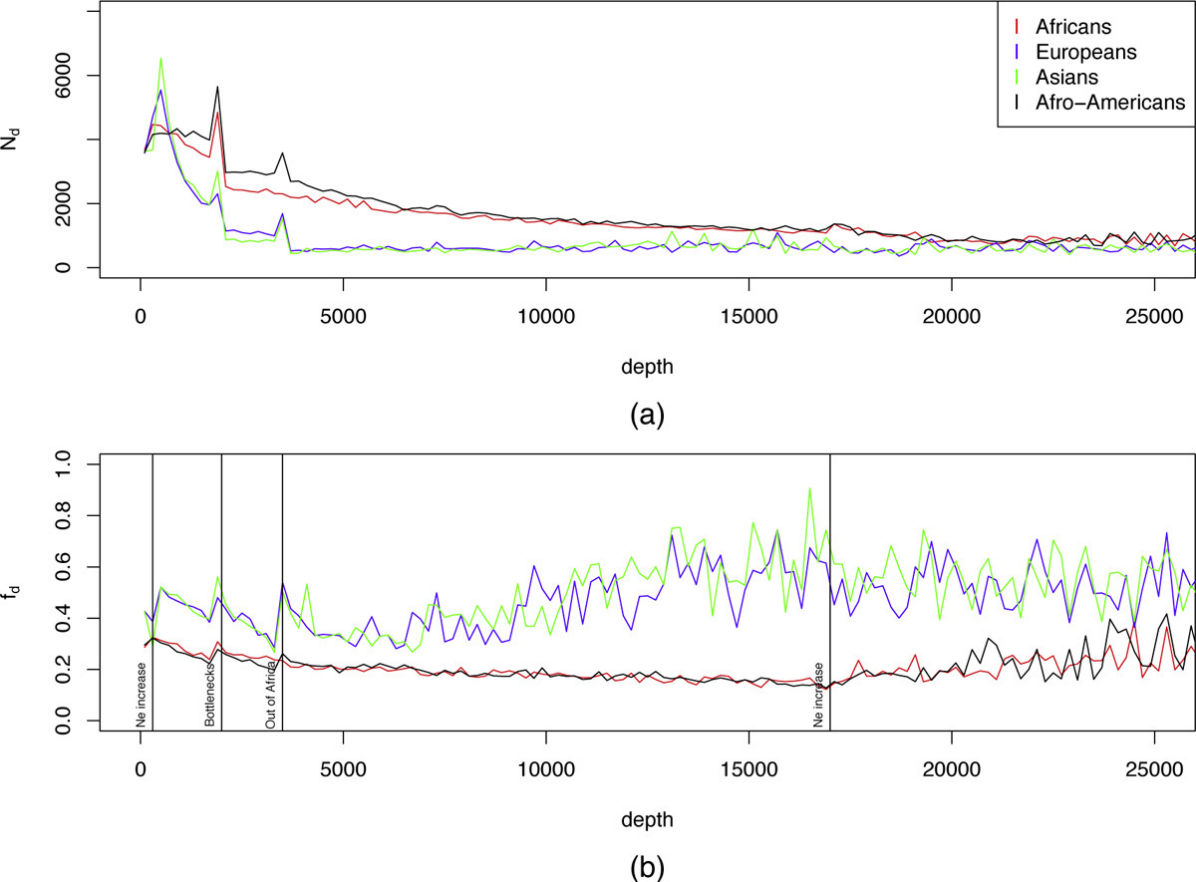
**The values of *N_d _*and *f_d _*of relevant genetic events against depth**. The values of (a) *N_d _*and (b) *f_d _*of relevant genetic events against depth, for the four demographies. The data is over all sequence lengths with a mutation rate 1.5 × 10^-8 ^bp/gen, recombination rate 2.1 cM/Mb and sample size 60. The four vertical lines in (b) correspond to the four events incorporated in the simulator COSI: (1) increase in effective population size, (2) bottleneck event, (3) out of Africa event and (4) increase in effective population size.

See Figure 3(a) for the plot of density against depth for each of the demographies. We find two broad categories of the profiles: (1) the African and the Afro-American demographies though fairly distinct at some points in the profile. (2) The European and Asian demographies are much more similar than the former pair. In general, the Asian and European demographies show a lower density of nodes than the other two demographies. However, the estimable density of the Asian and European demographies exceeds that of the other populations, under most parameter settings (see Figure 3(b)). The rationale for this reversal in density and the estimable density values of the demographies is not immediately apparent. It is possible that the different population effective sizes (*N_e_*) for the demographies may play a role.

**Figure 4:**
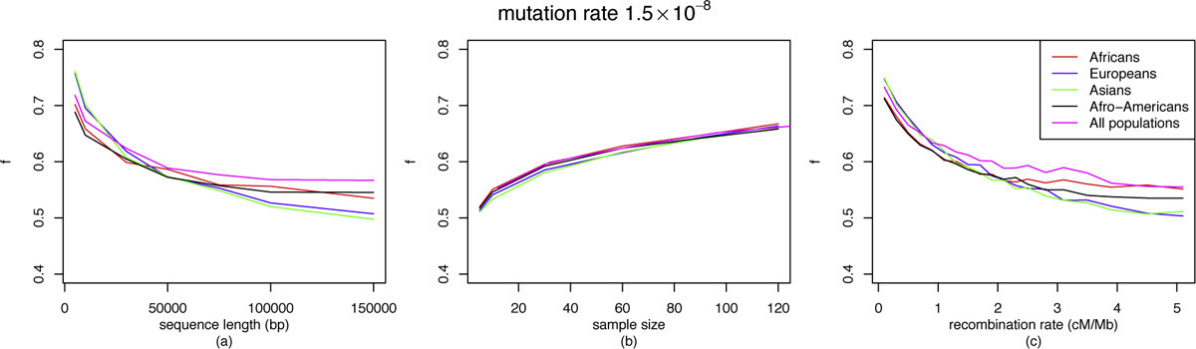
**Summary plots of *f***. Summary plots of *f *for each demography with mutation rate 1.5 × 10^-8 ^bp/gen for different values of (a) sequence length, (b) sample size and (c) recombination rate. For each value on the *x*-axis we consider the average of all possible values of the other parameters.

See Figure 4 for the effect of sequence length, sample size and recombination rate on *f*. Note that if there were no genetic exchange events in the ARG, this model leads to the generous overestimate of *f *= 1.0. Thus it is not very surprising to see a decreasing rate of recombination rate yielding increasing values of *f*, in each of the demography. With increase in sample size, *f *gradually increases but stabilizes around *f *= 0.65, suggesting that in all these demographies at least about 35% of the relevant genetic history is impenetrable. However, the most surprising observation comes from the experiments with different sample lengths. It turns out that *f *decreases with increasing sequence length. We observe that the reconstructable history of a segment *s *is actually smaller than the sum of the reconstructable histories of the subsegments of *s*. This is observed in each of the demographies in isolation, as well as when the demographies are combined into a single universal population.

## Conclusions and future directions

Reconstructability of common genetic history is a fundamental curiosity in the study of populations. While the population evolution models mature and the algorithms get more sophisticated, what fraction of the common and relevant genetic history of populations continues to be elusive? We present a framework that enables such an exploration. This is based on the random topological structure, the ARG and a method-independent (information-theoretic) structure called the minimal descriptor. This is applied to different demographics in a simulation setting. The most surprising observation is that the sum of the reconstructible history of each of the chromosomal segments, *s*_1_, *s*_2_, ..., *s_m_*, is indeed larger than the reconstructible history of the single segment composed of these segments. This appears to be a universal property, holding in all the demographies tested. Also, irrespective of the sample size, we observe that at least one-third of the population genetic history is impenetrable, in all the demographies.

The framework also opens up possible new directions of investigation. Assume that the characteristics of a population can be derived, say from the linkage disequilibrium landscape and other characteristics of observed extant individuals. Then, can such a generator be used to answer the "best-practice" questions about the population: what is the (1) optimal sample size and (2) optimal sequence length for the most informative analysis.

## Competing interests

The authors declare that they have no competing interests.

## Authors' contributions

FU implemented the estimability algorithm. MP and FU carried out the experiments and the analysis. LP designed the study. LP and FU wrote the paper.

## Declarations

The publication costs for this article were funded by the corresponding author's institution.

This article has been published as part of *BMC Genomics *Volume 14 Supplement 1, 2013: Selected articles from the Eleventh Asia Pacific Bioinformatics Conference (APBC 2013): Genomics. The full contents of the supplement are available online at http://www.biomedcentral.com/bmcgenomics/supplements/14/S1.

## Supplementary Material

Additional file 1 **Supplementary Material**. Click here for file

## References

[B1] Griffiths RC Marjoram P An ancestral recombinations graph Progress in Population Genetics and Human Evolution (P Donnelly and S Tavare Eds) IMA vols in Mathematics and its Applications 1997 87 257 270

[B2] Parida L Heath L, Ramakrishnan N Graph model of coalescence with recombinations Problem Solving Handbook in Computational Biology and Bioinformatics 2010 85 100

[B3] Parida L Palamara P Javed A A minimal descriptor of an ancestral recombinations graph BMC Bioinformatics 2011 12 S6 21342589 10.1186/1471-2105-12-S1-S6 PMC3044314

[B4] Parida L Nonredundant representation of ancestral recombinations graphs Methods Mol Biol 2012 856 315 332 10.1007/978-1-61779-585-5_13 22399465

[B5] Schaffner S Foo C Gabriel S Reich D Daly M Altshuler D Calibrating a coalescent simulation of human genome sequence variation Genome Res 2005 15 1576 1583 10.1101/gr.3709305 16251467 PMC1310645

[B6] Li H Durbin R Inference of human population history from individual whole-genome sequences Nature 2011 475 493 496 10.1038/nature10231 21753753 PMC3154645

[B7] Pickrell J Coop G Novembre J Kudaravalli S Li J Absher D Srinivasan B Barsh G Myers R Feldman M Pritchard J Signals of recent positive selection in a worldwide sample of human populations Genome Research 2009 19 826 837 10.1101/gr.087577.108 19307593 PMC2675971

[B8] Sabeti P Varilly P Fry B Lohmueller J Hostetter E Cotsapas C Xie X Byrne E Mccarroll S Gaudet R Schaffner S Lander E Consortium TIH Genome-wide detection and characterization of positive selection in human populations Nature 2007 449 7164 913 918 10.1038/nature06250 17943131 PMC2687721

[B9] Javed A Pybus M Melè M Utro F Bertranpetit J Calafell F Parida L IRiS: construction of ARG network at genomic scales Bioinformatics 2011 27 2448 2450 10.1093/bioinformatics/btr423 21765095

[B10] Sachidanandam R Weissman D Schmidt S Kakol J Stein L Marth G Sherry S Mullikin J Mortimore B Willey D Hunt S Cole C Coggill P Rice C Ning Z Rogers J Bentley D Kwok P Mardis E Yeh R Schultz B Cook L Davenport R Dante M Fulton L Hillier L Waterston R McPherson J Gilman B Schaffner S Van Etten W Reich D Higgins J Daly M Blumenstiel B Baldwin J Stange-Thomann N Zody M Linton L Lander E Altshuler D International SNP Map Working Group A map of human genome sequence variation containing 1.42 million single nucleotide polymorphisms Nature 2001 409 928 933 10.1038/35057149 11237013

[B11] Kong A Gudbjartsson D Sainz J Jonsdottir G Gudjonsson S Richardsson B Sigurdardottir S Barnard J Hallbeck B Masson G Shlien A Palsson S Frigge M Thorgeirsson T Gulcher J Stefansson K A high-resolution recombination map of the human genome Nature genetics 2002 31 241 247 12053178 10.1038/ng917

[B12] McVean G Myers S Hunt S Deloukas P Bentley D Donnelly P The fine-scale structure of recombination rate variation in the human genome Science 2004 304 581 4 10.1126/science.1092500 15105499

[B13] Parida L Ancestral recombinations graph: a reconstructability perspective using random-graphs framework Journal of Computational Biology 2010 17 1345 1350 10.1089/cmb.2009.0243 20976875

