# Supplementary material for: Sum of parts is greater than the whole: inference of common genetic history of populations
Source: BMC Genomics. 2013 Jan 21;14(Suppl 1):S10. doi: 10.1186/1471-2164-14-S1-S10 (PMC3549811; doi:10.1186/1471-2164-14-S1-S10)
Supplement: Additional file 1 — Supplementary Material. [file 1471-2164-14-S1-S10-S1.pdf]

Additional File 1 - Sum of parts is greater than the whole: inference of common genetic history of populations

Filippo Utro [1], Marc Pybus [2], Laxmi Parida*[1]

[1]Computational Genomics, IBM T J Watson Research, Yorktown, USA.
[2]Institute of Evolutionary Biology (CSIC-UPF), Dr. Aiguader 88, 08003 Barcelona, Spain.

Email: {futro,parida}@us.ibm.com; marc.pybus@upf.edu;

*Corresponding author

Outline

In this supplementary, we show in Figure S1 a complete example of the process and the results obtained for all parameters set-up considered in the papers.

Supplementary Material

Invariance of estimable fraction f over mutation rates

Figures S2 and S3 (see also Figure 4 in the main manuscript) shows the values of f for different values of sequence length, sample size and recombination rates in the simulations. The different demographies are shown together for comparison. The different mutation rates do not appear to affect the plots significantly.

Estimable fraction f within demographies

Figures S4-S8 shows the boxplots of f for the 10 replicates of each experiment. These were generated with the `boxplot` command in the R language [14]. For the convenience of the reader, we quote the boxplot definition here: *The rectangle shows the interquartile range (IQR); it goes from the first quartile (the 25th percentile) to the third quartile (the 75th percentile). The whiskers go from the minimum value to the maximum value unless the distance from the minimum value to the first quartile is more than 1.5 times the IQR. In that case the whisker extends out to the smallest value within 1.5 times the IQR from the first quartile. A similar rule is used for values larger than 1.5 times IQR from the third quartile. A special symbol shows the values, called outliers, which are smaller or larger than the whiskers.* We observe that:

1. with increase in the sequence length in the simulations, f decreases. It is interesting to see how different demographies seem to affect this trend. Populations with higher population effective sizes seem to have a lower reduction.

2. with increase in the sample size in the simulations, f increases and stabilizes around $f = 0.65$. Also, different demographies do not seem to affect this trend.

3. with increase in the the recombination rate in the sample simulations, f decreases, as expected.

Figures S9-S12 shows the summary plot of the four demographies used in our experiments.

```
0.000000 create_pop pop: 1 size: 0
0.000000 change_size pop: 1 size: 50
0.000000 ADD node: 0 pop: 1
0.000000 ADD node: 1 pop: 1
0.000000 ADD node: 2 pop: 1
0.000000 ADD node: 3 pop: 1
0.000000 ADD node: 4 pop: 1
0.000000 ADD node: 5 pop: 1
0.000000 ADD node: 6 pop: 1
0.000000 ADD node: 7 pop: 1
0.000000 ADD node: 8 pop: 1
0.000000 ADD node: 9 pop: 1
2.557704 C 3 8 -> 10 pop: 1
3.565347 C 1 5 -> 11 pop: 1
11.247099 C 9 10 -> 12 pop: 1
12.834033 C 12 11 -> 13 pop: 1
14.180100 R 7 -> 14 15 1 0.722288
26.297124 R 6 -> 16 17 1 0.422012
27.647837 C 17 0 -> 18 pop: 1
33.646579 C 18 13 -> 19 pop: 1
35.574414 C 15 4 -> 20 pop: 1
35.902590 C 14 19 -> 21 pop: 1
54.288687 C 20 21 -> 22 pop: 1
89.790844 R 16 -> 23 24 1 0.368156
92.466856 C 23 2 -> 25 pop: 1
111.756751 C 25 22 -> 26 pop: 1
111.756751 D 26 -> 28 | 27 0.000000 0.368156
111.756751 D 28 -> 30 | 29 0.422012 0.722288
111.756751 D 30 -> 32 | 31 0.722288 1.000000
282.316325 C 24 32 -> 33 pop: 1
```

(a)

```
paramfile: params
A 1 10
params: length 250000 mu 0.000000050
L 250000
  pos  anc1 anc2  freq  nodes...
> [0.000000, 0.368156]  time 470  E[muts] = 2.163020 (1)
M 0.15434826  10 3  1   3
> [0.368156, 0.053856]  time 830  E[muts] = 0.559046 (1)
M 0.39862645  18 0  1   0
> [0.422012, 0.300276]  time 405  E[muts] = 1.520911 (1)
M 0.69671637  26 22  9   0 1 3 4 5 6 7 8 9
> [0.722288, 0.277712]  time 404  E[muts] = 1.405484 (2)
M 0.79979988  17 6  1   6
M 0.93621548  20 4  1   4
```

(b)

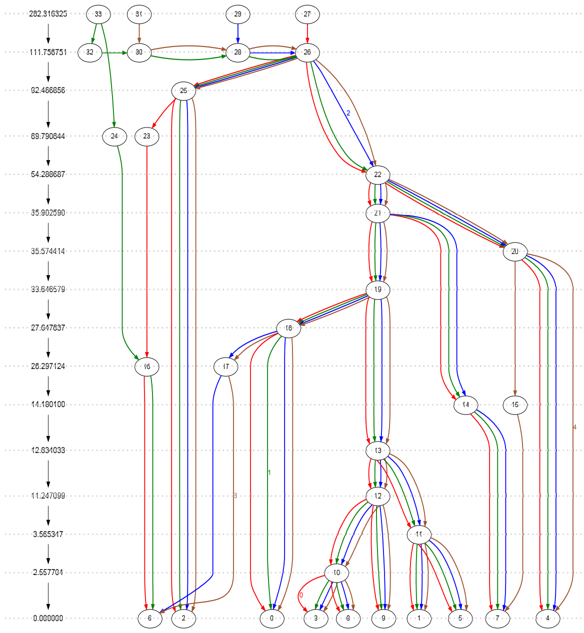

(c)

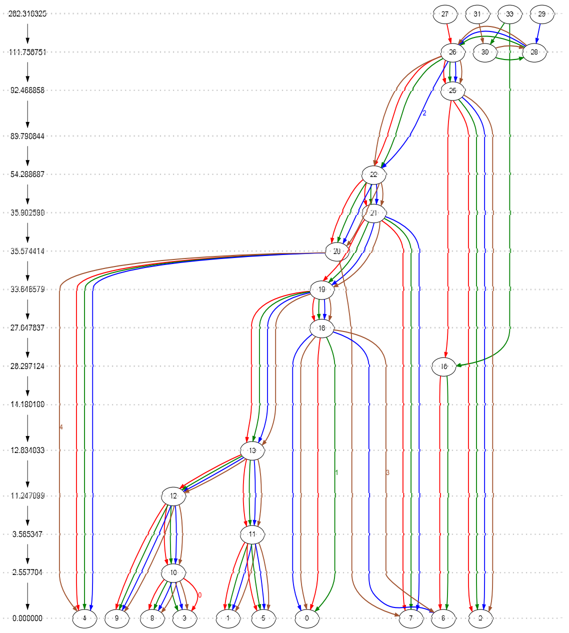

(d)

Figure S1: A complete example: (a) The log files produced as output of COSI. (b) The segment files produced as output of COSI. (c) The ARG derived from (a) and (b). (d) The minimal descriptor of (c).

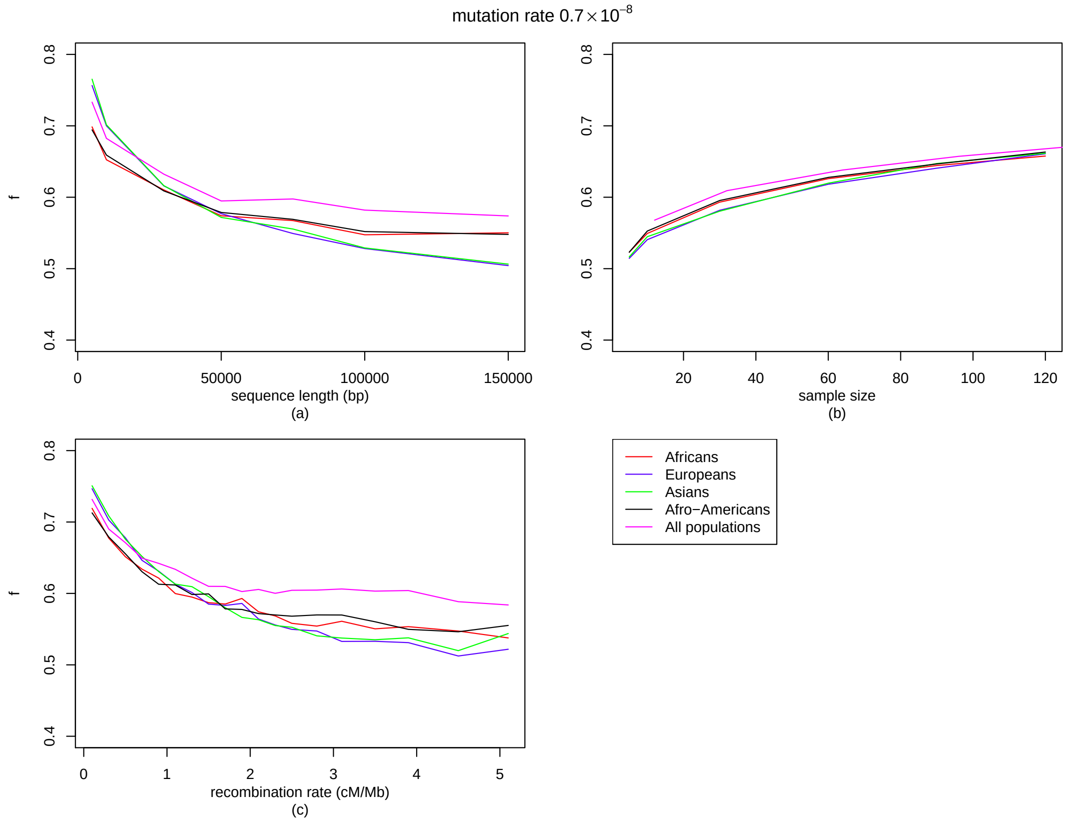

Figure S2: Summary plots of f for each demography with mutation rate 0.7×10^{-8} bp/gen. For each value on the x-axis we average of all possible values of the other parameters.

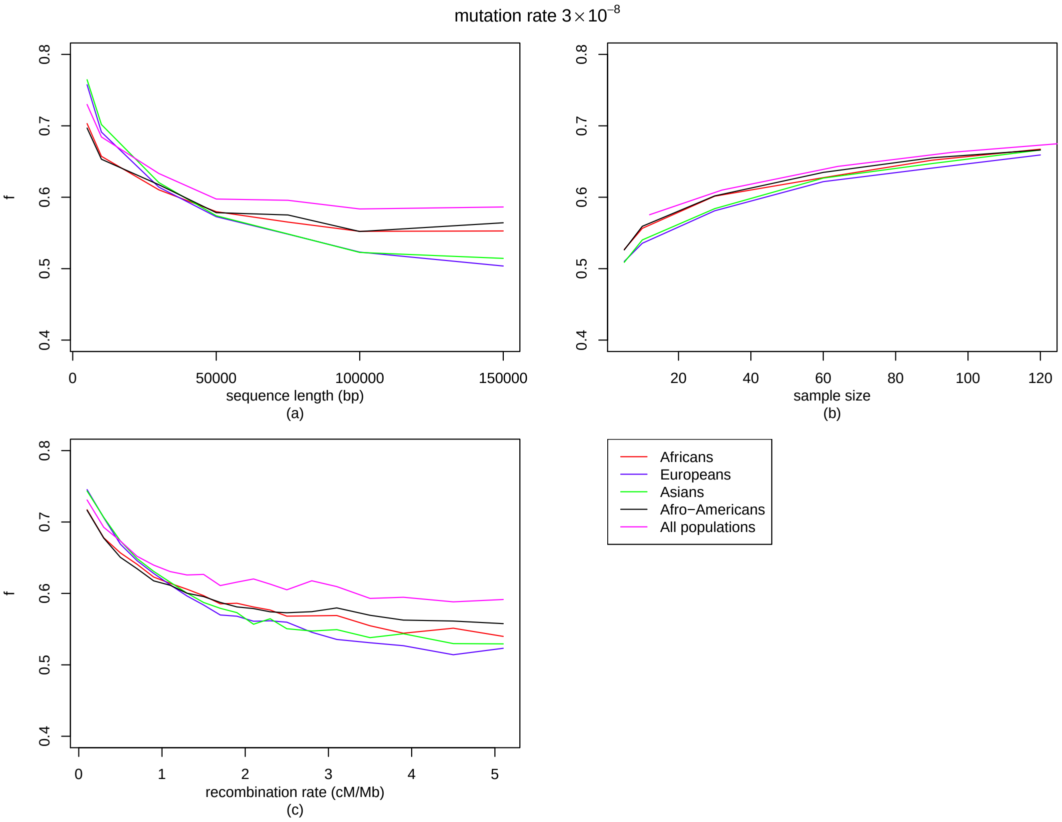

Figure S3: Summary plots of f for each demography with mutation rate 3.0×10^{-8} bp/gen. For each value on the x-axis we average of all possible values of the other parameters.

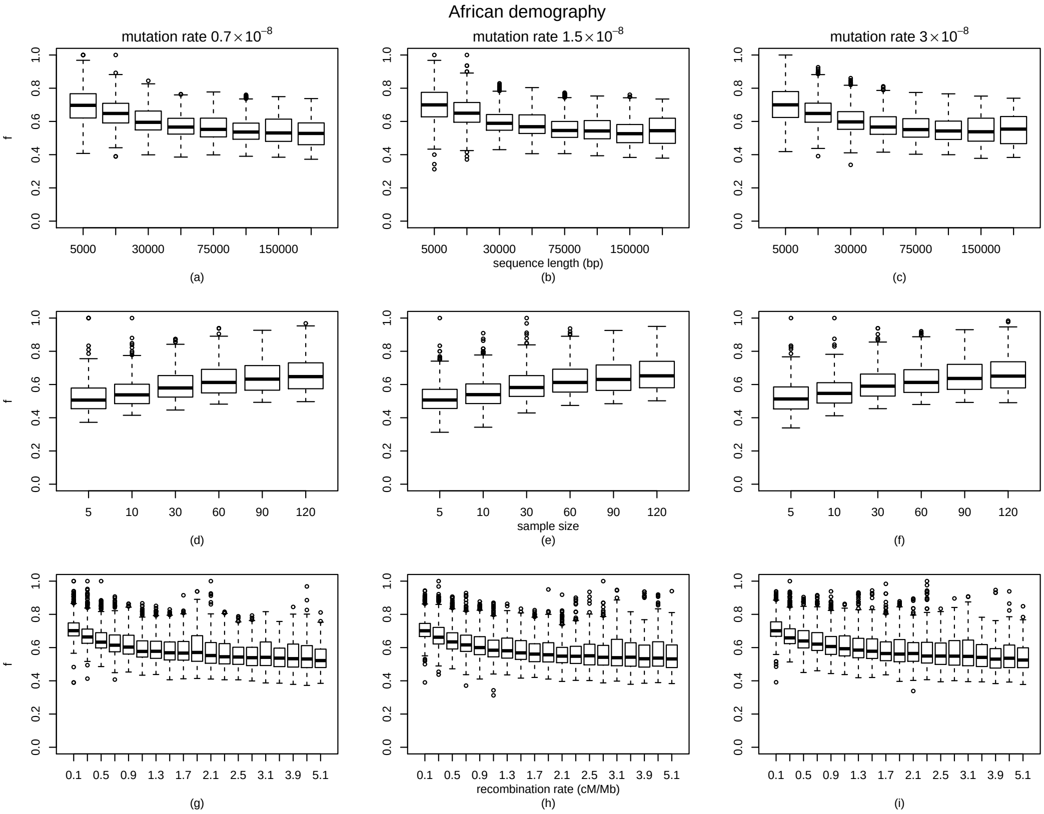

Figure S4: Box plots of the f for the Africans demography for different experimental set-up. Each column show the results for the different values of mutation rate. Each row shows the results for sequence length, sample size and recombination rate, respectively. For each value on the x-axis we consider the average of all possible values of the other parameters.

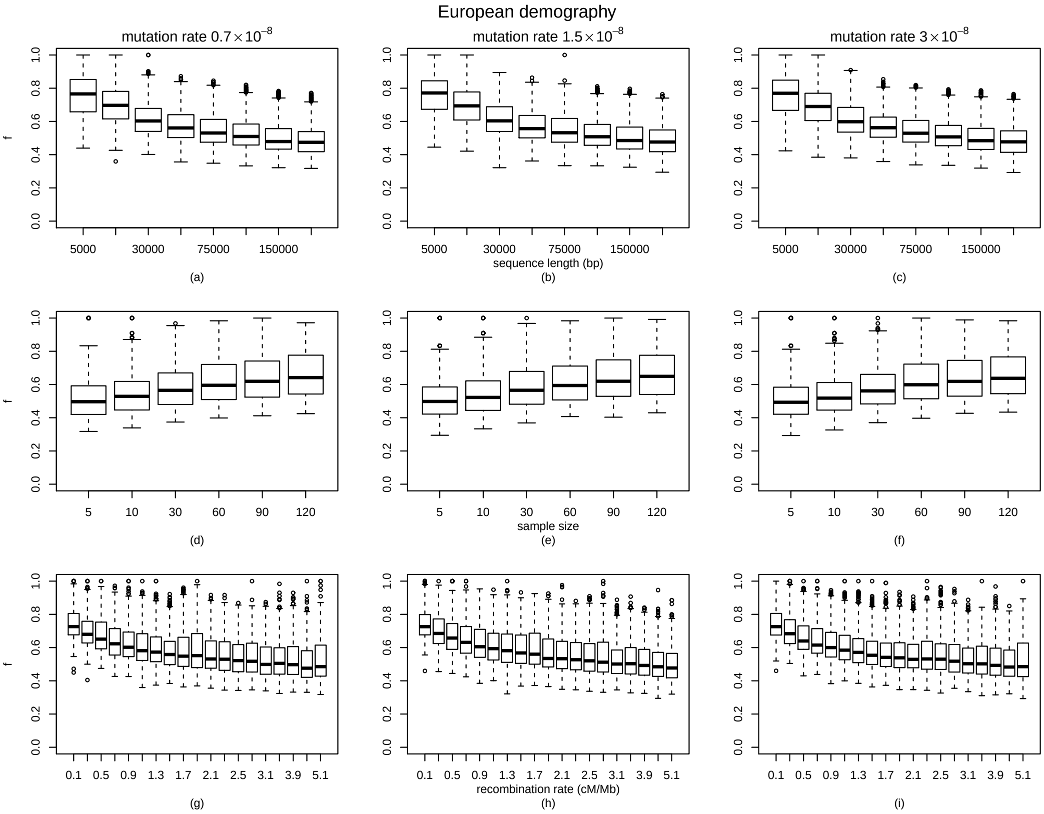

Figure S5: Box plots of the f for the European demography for different experimental set-up. Each column show the results for the different values of mutation rate. Each row shows the results for sequence length, sample size and recombination rate, respectively For each value on the x-axis we consider the average of all possible values of the other parameters.

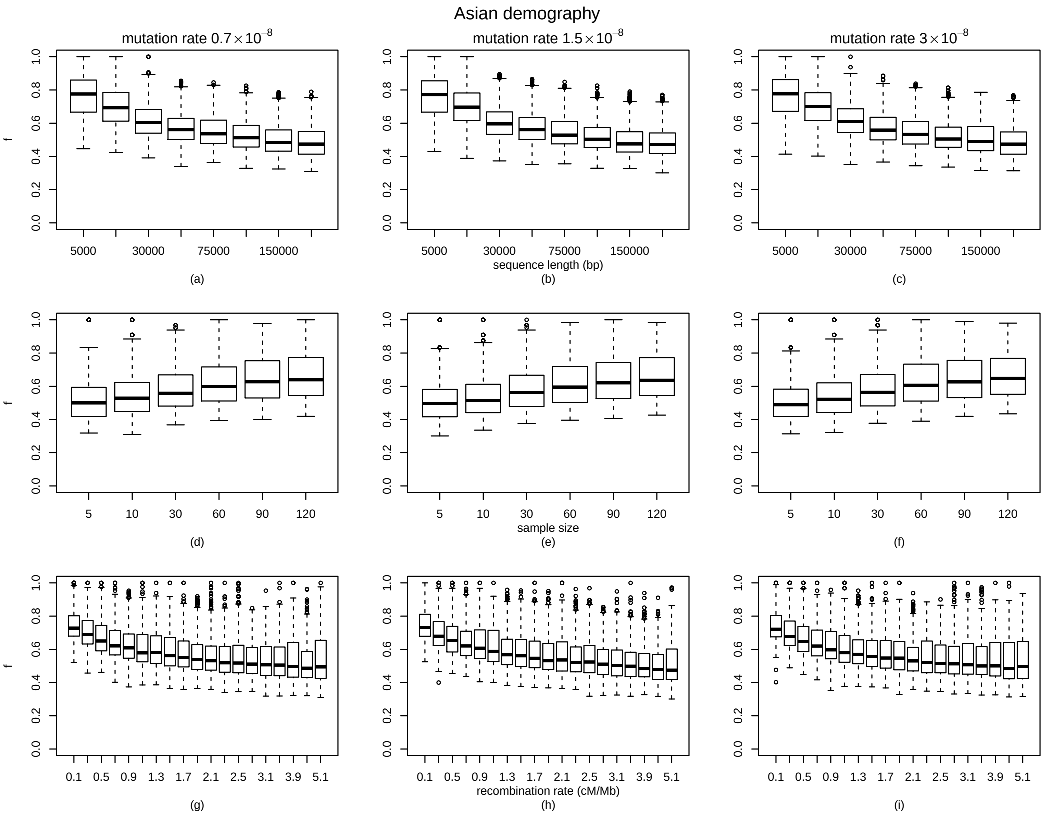

Figure S6: Box plots of the f for the Asian demography for different experimental set-up. Each column show the results for the different values of mutation rate. Each row shows the results for sequence length, sample size and recombination rate, respectively For each value on the x-axis we consider the average of all possible values of the other parameters.

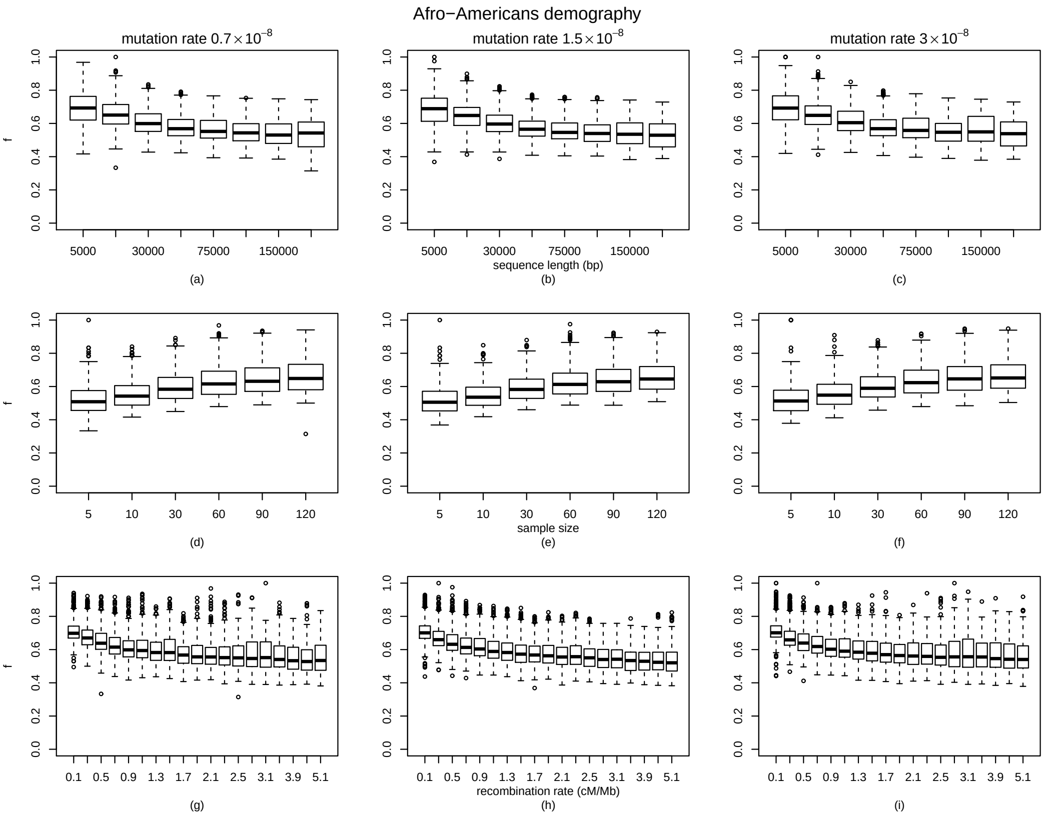

Figure S7: Box plots of the f for the Afro-American demography for different experimental set-up. Each column show the results for the different values of mutation rate. Each row shows the results for sequence length, sample size and recombination rate, respectively For each value on the x-axis we consider the average of all possible values of the other parameters.

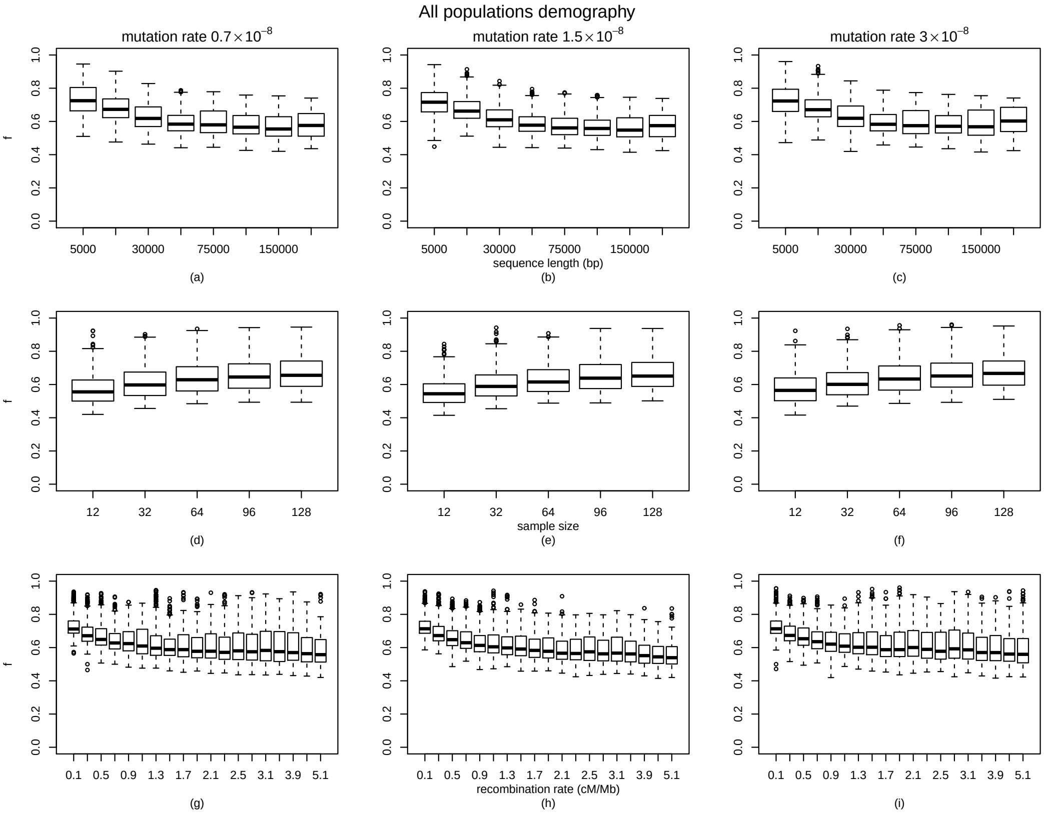

Figure S8: Box plots of the f for the whole demography for different experimental set-up. Each column show the results for the different values of mutation rate. Each row shows the results for sequence length, sample size and recombination rate, respectively. For each value on the x-axis we consider the average of all possible values of the other parameters.

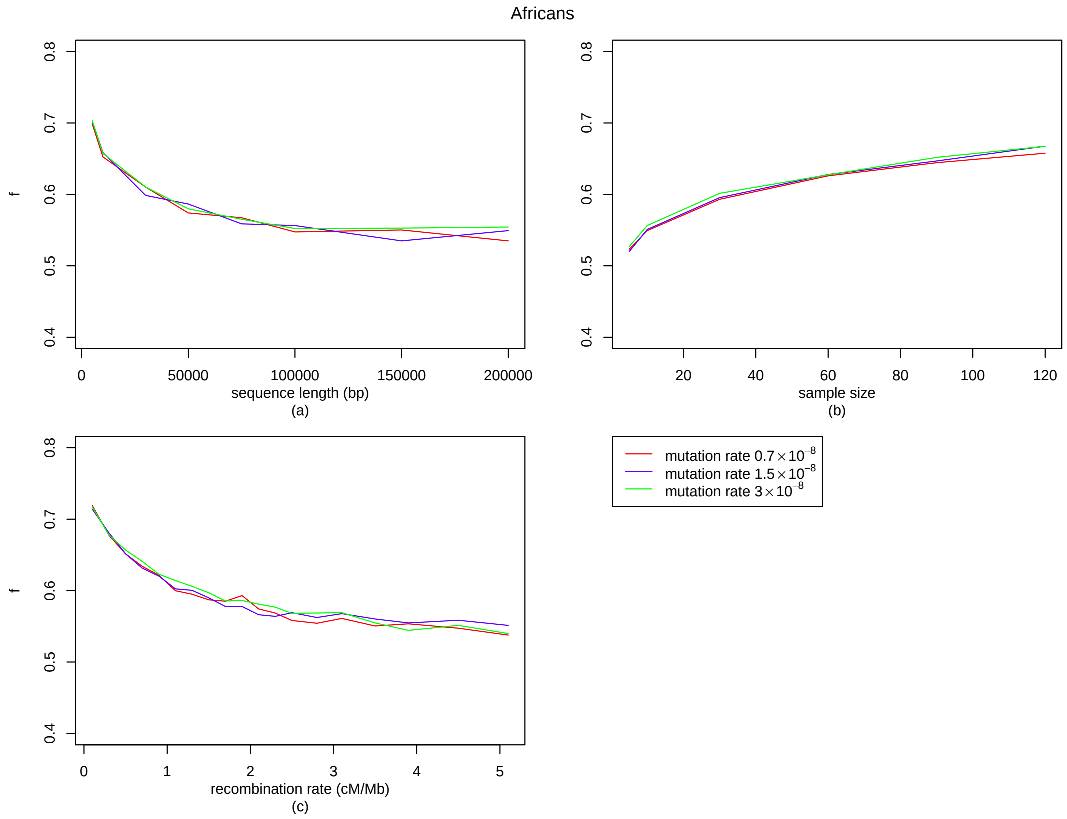

Figure S9: Summary plots of the f for the Africans demography for different values of (a) sequence length, (b) sample size and (c) recombination rate. For each value on the x-axis we consider the average of all possible values of the other parameters.

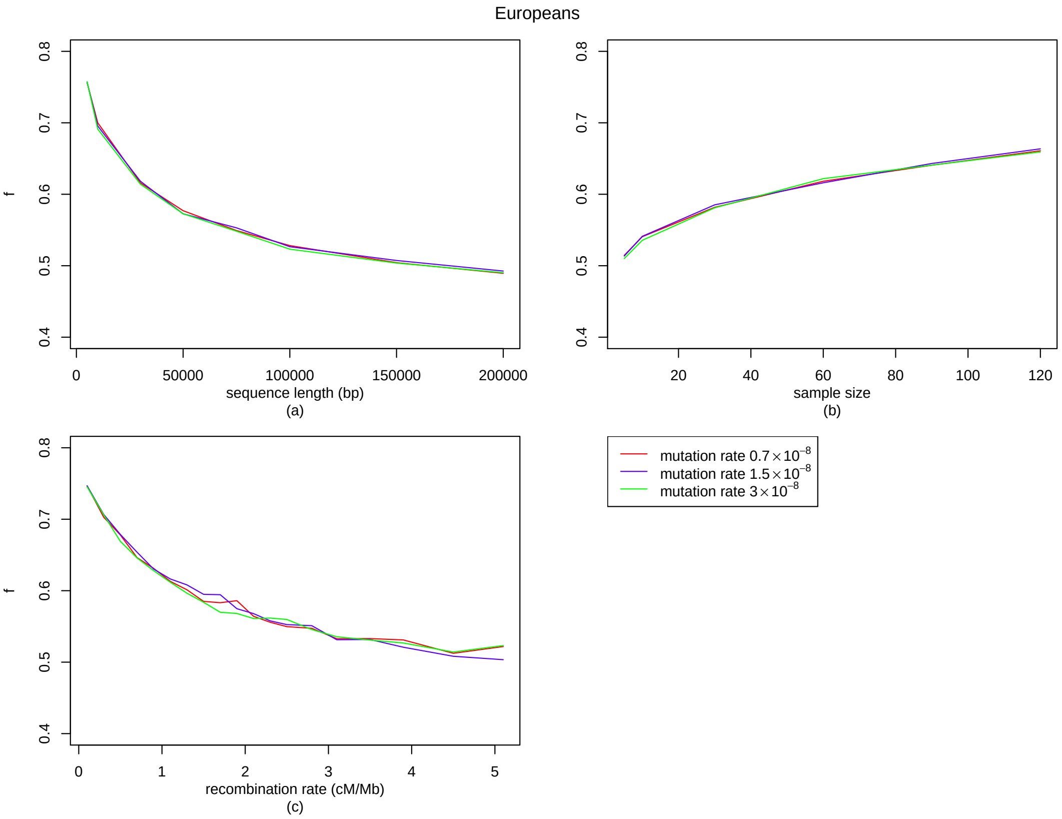

Figure S10: Summary plots of the f for the European demography for different values of (a) sequence length, (b) sample size and (c) recombination rate. For each value on the x-axis we consider the average of all possible values of the other parameters.

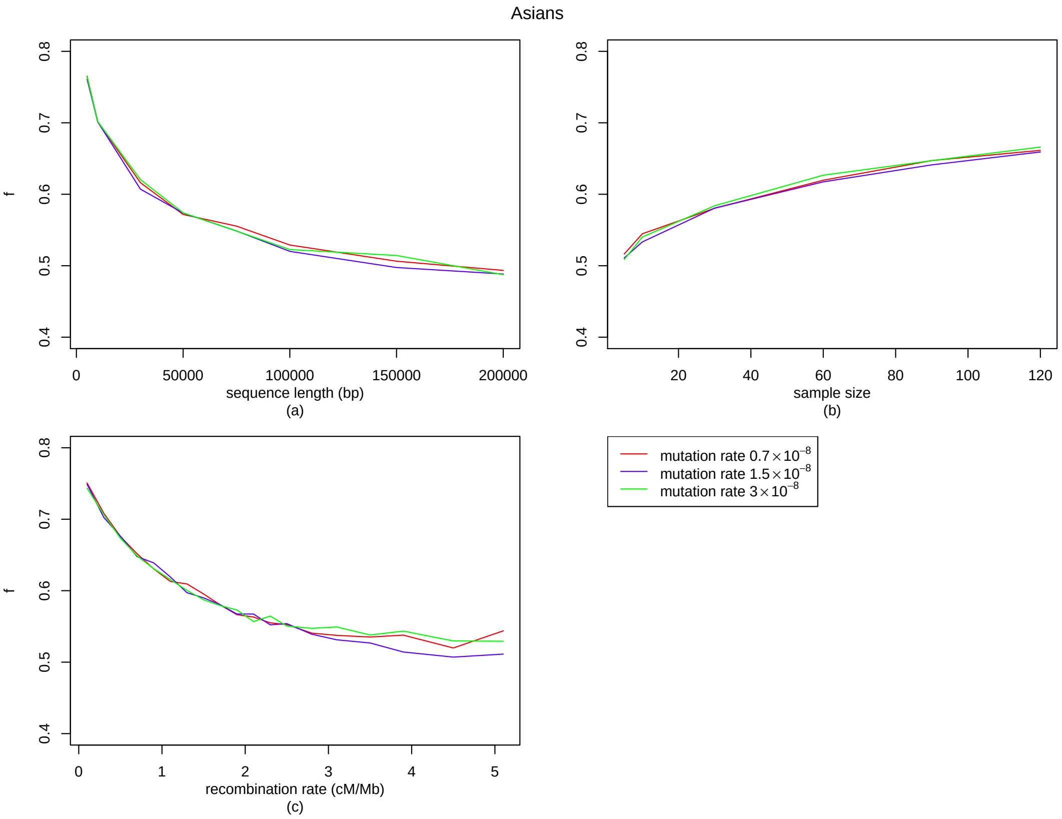

Figure S11: Summary plots of the f for the Asian demography for different values of (a) sequence length, (b) sample size and (c) recombination rate. For each value on the x-axis we consider the average of all possible values of the other parameters.

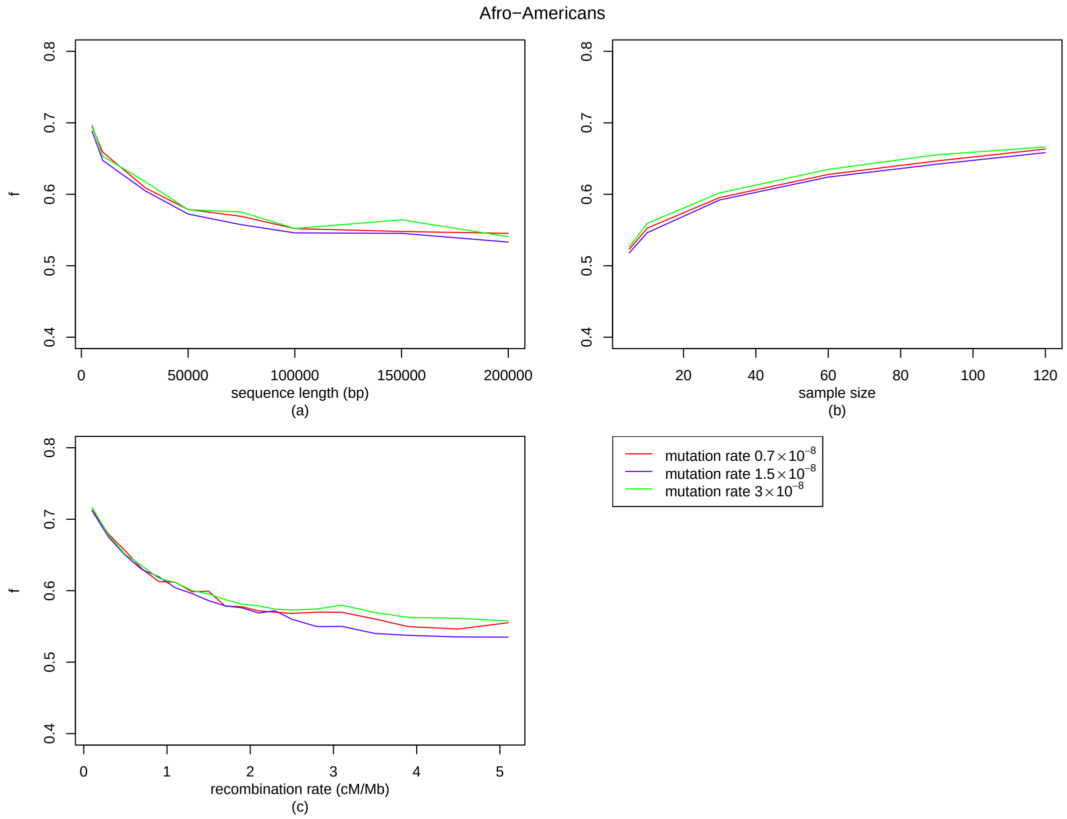

Figure S12: Summary plots of the f for the Afro-American demography for different values of (a) sequence length, (b) sample size and (c) recombination rate. For each value on the x-axis we consider the average of all possible values of the other parameters.
